# Supplementary material for: Clinical Differentiation of Severe Fever with Thrombocytopenia Syndrome from Japanese Spotted Fever
Source: Viruses. 2022 Aug 18;14(8):1807. doi: 10.3390/v14081807 (PMC9415593; doi:10.3390/v14081807)
Supplement: Supplementary file 1 [file viruses-14-01807-s001.zip › viruses-1819855-supplementary.pdf]

Supplementary materials

Table S1. Outcomes, complications and treatments of patients with JSF and SFTS

	all (n = 61)	SFTS (n = 23)	JSF (n = 38)	P value
Outcomes				
In-hospital death	6 (9.8)	6 (26.1)	0 (0.0)	0.002 †
Length of hospital stay (days)	23.1 ± 39.3	34.5 ± 57.7	15.9 ± 18.1	0.015 †
ICU admission	10 (16.4)	7 (30.4)	3 (7.9)	0.032 †
Complications				
Secondary bacterial infection	8 (13.1)	6 (26.1)	2 (5.3)	0.044 †
Secondary fungal infection	5 (8.2)	4 (17.4)	1 (2.6)	0.062
meningitis	2 (3.3)	1 (4.4)	1 (2.6)	1.000
Hemophagocytic lymphohistiocytosis (HLH)	11/60 (18.3)	11/23 (47.8)	0/37 (0.0)	< .0001 †
DIC	32 (52.5)	16 (69.6)	16 (42.1)	0.063
Treatments				
Tetracycline	55 (90.2)	17 (73.9)	38 (100.0)	0.002 †
Quinolone	27 (44.3)	15 (65.2)	12 (31.6)	0.016 †
Favipiravir	5 (8.2)	5 (21.7)	-	-
Ribavirin	2 (3.3)	2 (8.7)	-	-
Corticosteroids	11 (18.0)	9 (39.1)	2 (5.3)	0.002 †
G-CSF	7 (11.5)	7 (30.4)	0 (0.0)	0.001 †
Intravenous immunoglobulin	8/37 (13.3)	6/23 (26.1)	2/60 (5.4)	0.045 †
Thrombomodulin	22 (36.1)	12 (52.2)	10 (26.3)	0.056
Use of vasopressors	12 (19.7)	7 (30.4)	5 (13.2)	0.182
Oxygen therapy	11/60 (18.3)	7/23 (30.4)	4/37 (10.8)	0.086
Mechanical ventilation	8/60 (13.3)	6/22 (27.3)	2/38 (5.3)	0.042 †
Hemodialysis	3 (4.9)	2 (8.7)	1 (2.6)	0.551
CHDF	4 (6.6)	3 (13.0)	1 (2.6)	0.146
Plasmapheresis	3 (4.9)	2 (8.7)	1 (2.6)	0.551

Data are presented as the number of patients (%) or mean ± SD. Abbreviations: SFTS, severe fever with thrombocytopenia syndrome; JSF, Japanese spotted fever; DIC, disseminated intravascular coagulation; ICU, intensive care unit; G-CSF, granulocyte colony-stimulating factor; CHDF, continuous hemodiafiltration. † Significant difference level: 0.05.

Table S2. Sensitivity, specificity, positive predictive value and negative predictive value for differentiating SFTS from JSF

	AUC	Sensitivity	Specificity	PPV	NPV
Altered mental status	0.699	0.609	0.790	0.636	0.769
Diarrhea	0.709	0.524	0.895	0.733	0.773
Absence of skin rash	0.826	0.571	1.000	1.000	0.826
Absence of tick bite	0.730	0.455	0.889	0.750	0.780
WBC < 4000 (/μL)	0.978	0.957	1.000	1.000	0.974
Neutrophil < 2042(/μL)	0.957	0.913	1.000	1.000	0.949
Atypical lymphocyte (≥3%)	0.609	0.217	1.000	1.000	0.673
Platelet < 64 ×10^3 (/μL)	0.743	0.696	0.790	0.667	0.811
AST ≥ 261 (IU/L)	0.756	0.565	0.947	0.867	0.783
ALT ≥ 57 (IU/L)	0.733	0.783	0.684	0.600	0.839
LDH ≥ 771 (IU/L)	0.783	0.684	1.000	1.000	0.792
CRP< 1.78 (mg/dL)	0.935	0.870	1.000	1.000	0.927
APTT ≥ 36.5 (s)	0.746	0.870	0.622	0.588	0.885

Abbreviations: SFTS, severe fever with thrombocytopenia syndrome; JSF, Japanese spotted fever; WBC, white blood cell; AST, aspartate aminotransferase; ALT, alanine transaminase; LDH, lactate dehydrogenase; CRP, C-reactive protein; APTT, activated partial thromboplastin; PPV, positive predictive value; NPV, negative predictive value.

Figure S1.

A. **B.**

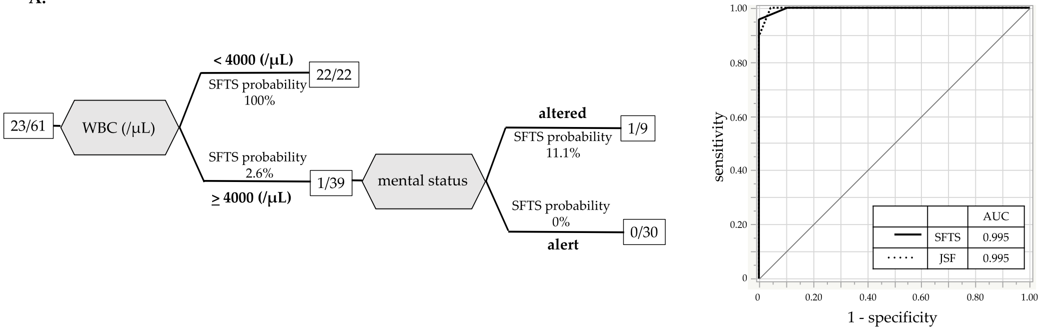

Figure S1. A. Decision tree classification of SFTS and JSF based on laboratory data and physical examination. The number in the square is the number of SFTS patients/total patients. **B.** Receiver operating characteristic curve analysis of differentiating SFTS and JSF by decision tree based on supplementary figure 1A.

Figure S2.

A. **B.**

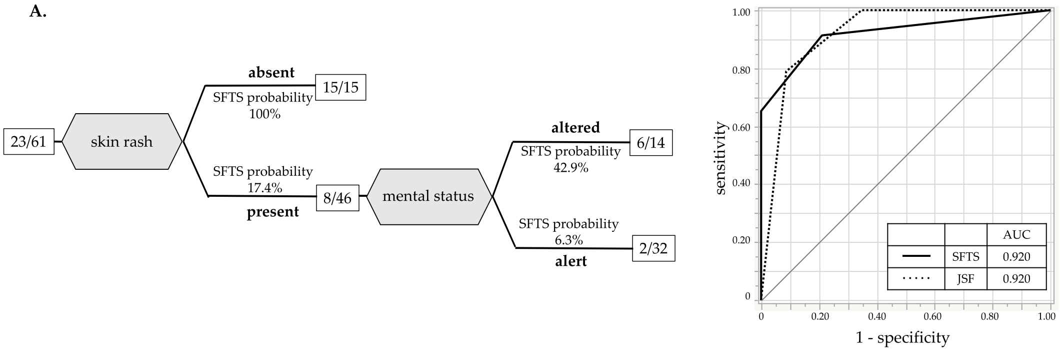

Figure S2. A. Decision tree classification of SFTS and JSF based on physical examination. The number in the square is the number of SFTS patients/total patients. **B.** Receiver operating characteristic curve analysis of differentiating SFTS and JSF by decision tree based on supplementary figure 2A.
